# Novel *Chlamydiaceae* Disease in Captive Salamanders

**DOI:** 10.3201/eid1806.111137

**Published:** 2012-06

**Authors:** An Martel, Connie Adriaensen, Sergé Bogaerts, Richard Ducatelle, Herman Favoreel, Sandra Crameri, Alex D. Hyatt, Freddy Haesebrouck, Frank Pasmans

**Affiliations:** Ghent University, Merelbeke, Belgium (A. Martel, C. Adriaensen, R. Ducatelle, H. Favoreel, F. Haesebrouck, F. Pasmans);; Waalre, the Netherlands (S. Bogaerts);; Commonwealth Scientific and Industrial Research Organisation, Geelong, Victoria, Australia (S. Crameri, A.D. Hyatt)

**Keywords:** Chlamydiales, Urodelans, Candidatus Amphibiichlamydia salamandrae, Neurergus sp., Salamandra sp., bacteria, salamander, amphibian, chlamydia

**To the Editor:** Although 2 major diseases of amphibians, chytridiomycosis and ranavirosis, have been relatively well studied, enigmatic amphibian disease and death not attributable to any of the known amphibian diseases frequently occur (*1*). We describe an apparently new disease in salamanders that is associated with a novel genus within the family *Chlamydiaceae*.

The salamanders seen in our clinic belonged to 1 of the following species: *Salamandra corsica*, the Corsican fire salamander (5 animals from 1 collection); *Neurergus crocatus*, the yellow spotted newt (11 animals from 3 collections); or *N. strauchii*, Strauch’s spotted newt (6 animals from 2 collections). All salamanders were captive bred; housed in breeding colonies in private collections in Elsloo and Eindhoven, the Netherlands, Munich, Germany, and Brugge, Belgium; and 1–3 years of age.

Disease was characterized by anorexia, lethargy, edema, and markedly abnormal gait. Mortality rate was 100%. Animals in these collections had no histories of disease. All animals were in good nutritional condition. Necropsy did not yield any macroscopic lesions. All animals had mild intestinal nematode or protozoan infections. Results of real-time PCRs for iridoviruses in liver and skin (*2*) or *Batrachochytrium dendrobatidis* fungus of skin (*3*) were negative for all animals.

We placed liver suspensions from the dead salamanders on Columbia agar with 5% sheep blood and tryptic soy agar and then incubated the samples up to 14 days at 20°C. No consistent bacterial growth was observed. Histologic examination of 2 Corsican fire salamanders and 1 yellow spotted newt revealed hepatitis in 1 of the Corsican fire salamanders and the yellow spotted newt. Hepatitis was characterized by high numbers of melanomacrophages and a marked infiltration of granulocytic leukocytes. Immunohistochemical staining for chlamydia (IMAGEN Chlamydia; Oxoid, Basingstone, UK) showed cell-associated fluorescently stained aggregates in liver tissue, suggestive of Chlamydiales bacteria. Transmission electron microscopic examination of the liver of a yellow spotted newt revealed intracellular inclusions containing particles matching the morphology of reticulate or elementary bodies of *Chlamydiaceae* (Technical Appendix).

A PCR (*4*) to detect the 16S rRNA of all Chlamydiales bacteria, performed on liver tissue samples from all animals, yielded positive results in all 5 Corsican fire salamanders; in 4/7, 1/3, and 1/1 yellow spotted newts; and in 4/5 and 1/1 Strauch’s spotted newts. For taxon identification, the 16S rRNA gene of the Chlamydiales bacteria was amplified and sequenced from the livers from 2 yellow spotted newts (1 from the collection in Elsloo, the Netherlands and 1 from the collection in Munich, Germany), 1 Strauch’s spotted newt, and 5 Corsican fire salamanders.

The sequences shared >90% nt identity with the 16S rRNA gene of *C. abortus* B577 (GenBank accession no. D85709) and therefore can be identified as a member of the family *Chlamydiaceae* (*5*). The closest 16S rRNA similarity (92%) was observed with *C. psittaci* strain CPX0308 (AB285329). The sequence obtained from all spotted newt species specimens was identical (GenBank accession no. JN392920) but differed slightly (1%) from that obtained from the fire salamander species specimens (GenBank accession no. JN392919). These sequence differences point to the existence of multiple strains with possible host adaptation.

We determined the phylogenetic position of the novel taxon, named *Candidatus* Amphibiichlamydia salamandrae (Technical Appendix), identified by using neighbor-joining analysis with Kodon software (Applied Maths, Sint-Martens-Latem, Belgium). The novel Chlamydiales forms a distinct branch in the well-supported monophyletic clade with the genera *Chlamydia* and *Candidatus* Clavochlamydia salmonicola (family *Chlamydiaceae*) (Figure). Maximum parsimony and unweighted pair group with arithmetic mean analyses yielded cladograms with the same topology (results not shown). Previous reports of members of the family *Chlamydiaceae* in amphibians concerned species occurring in other vertebrate taxa as well: *C. psittaci*, *C. pneumoniae,* *C. abortus*, and *C. suis* (*6**–**10*). To our knowledge, this member of the family *Chlamydiaceae* has been seen in amphibians, but not in other vertebrate hosts. The 16S rRNA analysis showed this taxon to belong to a clade with *Candidatus* Clavochlamydia salmonicola, a taxon found in fish. The phylogenetic position of the novel taxon in the family *Chlamydiaceae* thus roughly reflects the phylogenetic relation between the host species, providing evidence for host–bacterium co-evolution in the family *Chlamydiaceae*.

**Figure:**
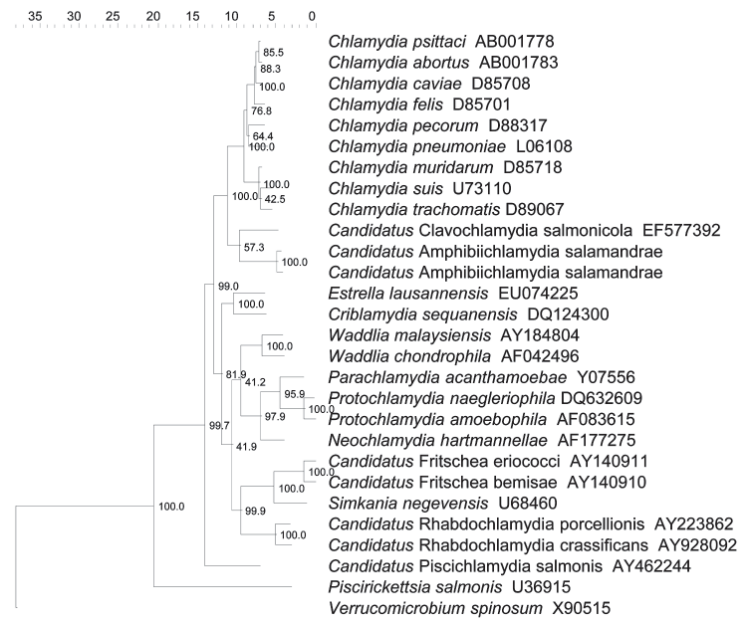
Topology of the novel amphibian *Chlamydiaceae* (*Candidatus* Amphibiichlamydia salamandrae) within the phylogenetic tree obtained by neighbor-joining and based on 16S rRNA gene data from representative species. Numbers show the percentage of times each branch was found in 1,000 bootstrap replicates. The tree has been rooted with *Verrucomicrobium spinosum* as outgroup. Scale bar indicates nucleotide substitutions per site.

Although the results obtained are not conclusive with regard to the pathogenic potential of this novel genus and species of Chlamydiales, we were not able to attribute the clinical signs to any known disease. We therefore suggest that we discovered a novel bacterial taxon with possible considerable impact on amphibian health.

## Supplementary Material

Technical Appendix Description of *Candidatus* Amphibiichlamydia salamandrae. Transmission electron micrograph of a liver section of a yellow spotted newt showing an intracellular vacuole containing elementary bodies and reticulate bodies of *Chlamydia*-like organisms.

## References

[1] Daszak P, Berger L, Cunningham AA, Hyat AD, Green DE, Speare R. Emerging infectious diseases and amphibian population declines. Emerg Infect Dis. 1999;5:735–48. 10.3201/eid0506.990601 10603206 PMC2640803

[2] Mao J, Hedrick RP, Chichar VB. Molecular characterization, sequence analysis, and taxonomic position of newly isolated fish iridoviruses. Virology. 1997;229:212–20. 10.1006/viro.1996.8435 9123863

[3] Boyle DG, Boyle DB, Olsen V, Morgan JAT, Hyatt AD. Rapid quantitative detection of Chytridiomycosis (*Batrachochytrium dendrobatidis*) in amphibian samples using real-time Taqman PCR assay. Dis Aquat Organ. 2004;60:141–8. 10.3354/dao060141 15460858

[4] Everett KDE. *Chlamydia* and Chlamydiales: more than meets the eye. Vet Microbiol. 2000;75:109–26. 10.1016/S0378-1135(00)00213-3 10889402

[5] Everett KDE, Bush RM, Andersen AA. Emended description of the order Chlamydiales, proposal of *Parachlamydiaceae* fam. nov. and *Simkaniaceae* fam. nov., each containing one monotypic genus, revised taxonomy of the family *Chlamydiaceae*, including a new genus and five new species, and standards for the identification of organisms. Int J Syst Bacteriol. 1999;49:415–40. 10.1099/00207713-49-2-415 10319462

[6] Berger L, Volp K, Mathews S, Speare R, Timms P. *Chlamydia pneumoniae* in a free-ranging giant barred frog (*Mixophyes iterates*) from Australia. J Clin Microbiol. 1999;37:2378–80. 10364623 10.1128/jcm.37.7.2378-2380.1999 PMC85174

[7] Blumer C, Zimmermand DR, Weilenmann R, Vaughan L, Pospischil A. *Chlamydiae* in free-ranging and captive frogs in Switzerland. Vet Pathol. 2007;44:144–50. 10.1354/vp.44-2-144 17317791

[8] Howerth EW. Pathology of naturally-occurring chlamydiosis in African clawed frogs (*Xenopus laevis*). Vet Pathol. 1984;21:28–32. 6710808 10.1177/030098588402100105

[9] Newcomer CE, Anver MR, Simmons JL, Wilcke RW, Nace GW. Spontaneous and experimental infections of *Xenopus laevis* with *Chlamydia psittaci.* Lab Anim Sci. 1982;32:680–6. 7162133

[10] Reed KD, Ruth GR, Meyer JA, Shukla SK. *Chlamydia pneumonia* infection in a breeding colony of African clawed frogs (*Xenopus tropicalis*). Emerg Infect Dis. 2000;6:196–9. 10.3201/eid0602.000216 10756157 PMC2640851

