# Supplementary material for: Novel Chlamydiaceae Disease in Captive Salamanders
Source: Emerg Infect Dis. 2012 Jun;18(6):1020–2. doi: 10.3201/eid1806.111137 (PMC3358148; doi:10.3201/eid1806.111137)
Supplement: Technical Appendix — Description of Candidatus Amphibiichlamydia salamandrae. Transmission electron micrograph of a liver section of a yellow spotted newt showing an intracellular vacuole containing elementary bodies and reticulate bodies of Chlamydia-like organisms. [file 11-1137-Techapp_1p.pdf]

Article DOI: http://dx.doi.org/10.3201/eid1806.111137

Novel *Chlamydiaceae* Disease in Captive Salamanders

Technical Appendix

Description of *Candidatus* Amphibiichlamydia salamandrae

Candidatus Amphibiichlamydia salamandrae [Amphibii chlamydia N.L. n. Amphibia name of host class; L. fem. n. *Chlamydia* name of bacterial taxon; N.L. fem. n. *Amphibiichlamydia Chlamydia* from an amphibian; salamandra'e. L. gen. n. salamandrae of a salamander]

The provisional taxon "*Candidatus* Amphibiichlamydia salamandrae" contains intracellular bacteria that infect salamanders of the genera *Neurergus* and *Salamandra* in freshwater or terrestrial environments. The 16S rRNA gene of *Candidatus* Amphibiichlamydia salamandrae has been deposited in the GenBank under accession nos. JN392920 and JN392919. The 16S rRNA gene shows phylogenetic affinity toward the family *Chlamydiaceae*.

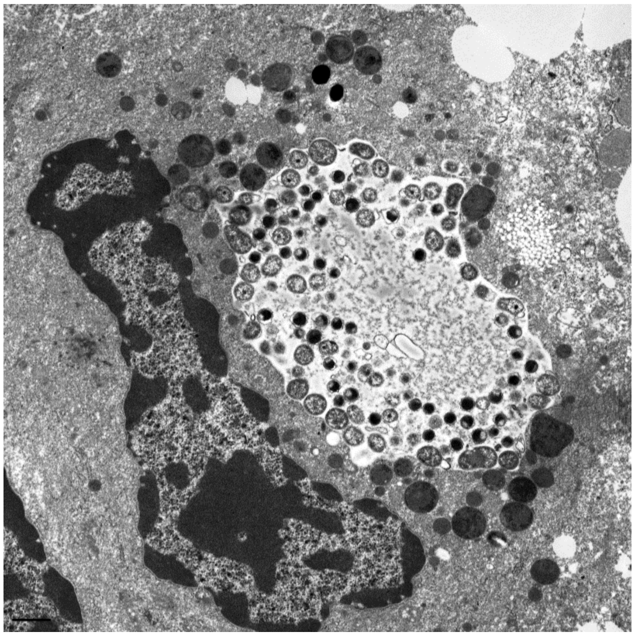

Technical Appendix Figure. Transmission electron micrograph of a liver section of a yellow spotted newt (*Neurergus crocatus*) showing an intracellular vacuole containing elementary bodies and reticulate bodies of *Chlamydia*-like organisms. Original magnification ×4,000.
